# P-2189. The Integration of Hepatitis C Treatment into Rural Syringe Services Programs via Telehealth: A Highly Effective Care Model

**DOI:** 10.1093/ofid/ofae631.2343

**Published:** 2025-01-29

**Authors:** Emily Hoff, Sherilyn Brinkley, Tracy Agee, Maria C Latimer, Patricia Tichnell, Jackie Bittner, Elizabeth Spradley, G Malik Burnett, Mark S Sulkowski, Oluwaseun Falade-Nwulia

**Affiliations:** Johns Hopkins, Baltimore, MD; Johns Hopkins University, Baltimore, Maryland; Johns Hopkins University, Baltimore, Maryland; Johns Hopkins University School of Medicine, Baltimore, Maryland; Johns Hopkins, Baltimore, MD; Johns Hopkins, Baltimore, MD; Maryland Department of Health, Baltimore, Maryland; Maryland Department of Health, Baltimore, Maryland; Johns Hopkins University School of Medicine, Baltimore, Maryland; Johns Hopkins University, Baltimore, Maryland

## Abstract

**Background:**

Direct acting antivirals (DAA) effectively cure Hepatitis C (HCV) infection; however, uptake among people who inject drugs (PWID) is low due to social determinants of health and healthcare system barriers. We present a telehealth model of HCV care integration into syringe services programs (SSPs).**Figure 1.** The HCV Care Continuum through ACCESS Telehealth, a program to integrate hepatitis C and opioid use disorder care into rural Maryland syringe services programs.

**Figure:**
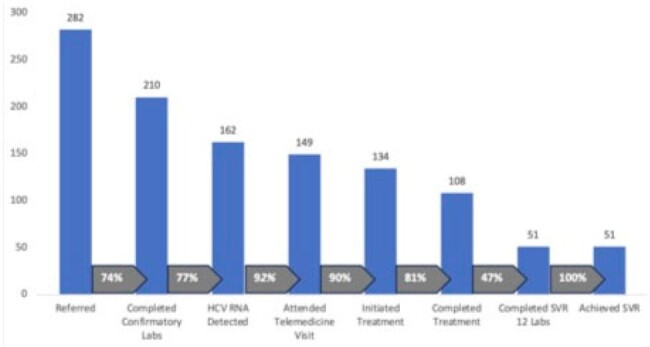
Note: At time of analysis 6 were pending insurance approval, 15 patients were still on treatment, and 10 had not yet reached the time point for SVR 12 assessment.

**Methods:**

The Johns Hopkins Viral Hepatitis Center implemented ACCESS Telehealth in collaboration with the Maryland Department of Health Center for Harm Reduction Services. Partnering with individual SSPs, context evaluation, HCV testing workflows, referral pathways for HCV/Opioid Use Disorder (OUD) care, SSP staff training, and SSP-based private space for telemedicine-equipped encounters were established. Services began in August 2021 in three rural Maryland SSPs and expanded to two more in 2022. SSP staff refer clients to low threshold, HCV/OUD treatment. An off-site nurse case manager provides patient education and support, collaborating with SSP-based Certified Peer Recovery Specialists. Specialists offer HCV/OUD treatment at the SSPs via telemedicine. Appointment, prescription, and laboratory information were extracted from the electronic medical record. DAA initiation and completion was confirmed by pharmacy records. Sustained virologic response (SVR) was defined as HCV RNA < 15 IU/ml measured >12 weeks after treatment completion.

**Results:**

Of 282 patients scheduled for appointments, 84% (n=236) were white, 48% (134) were female, the median age of 39 years (interquartile range, 34-47) and 3% (11) were HIV-coinfected. Confirmatory labs were completed in 74% (210) of whom 23% (48) had spontaneously cleared HCV. Of 162 patients with detectable viremia, 92% (149) attended a provider visit, 90% (134) of those who attended a visit started treatment, 81% (108) of those who started treatment completed treatment, and 47% (51) of those who completed treatment had SVR labs drawn, of which 100% (51) achieved SVR (Figure 1). Overall, 45% (126) were prescribed buprenorphine.

**Conclusion:**

We demonstrate an effective model for HCV care integration into rural SSPs with high uptake and completion of curative HCV treatment ( >80%). However, more than half did not complete post-treatment evaluation to confirm HCV response. Strategies for care after HCV treatment are needed.

**Disclosures:**

Sherilyn Brinkley, MSN, CRNP, AbbVie: Honoraria|Gilead Sciences: Honoraria Mark S. Sulkowski, MD, AbbVie: Advisor/Consultant|Aligos Therapeutics: Advisor/Consultant|Gilead: Advisor/Consultant|GSK: Advisor/Consultant|GSK: Grant/Research Support|Janssen: Grant/Research Support|Precision Biosciences: Advisor/Consultant|Vir: Grant/Research Support|Virion: Advisor/Consultant Oluwaseun Falade-Nwulia, MBBS ,MPH, Abbvie Inc: Grant/Research Support|Gilead Sciences: Advisor/Consultant

